# Comparative Analysis of Roseoloviruses in Humans, Pigs, Mice, and Other Species

**DOI:** 10.3390/v11121108

**Published:** 2019-11-30

**Authors:** Joachim Denner, Tarin M. Bigley, Tuan L. Phan, Cosima Zimmermann, Xiaofeng Zhou, Benedikt B. Kaufer

**Affiliations:** 1 Robert Koch Institute, Robert Koch Fellow, 13352 Berlin, Germany; 2 Division of Rheumatology, Department. of Medicine, Washington University School of Medicine, St. Louis, MO 63110, USA; bigley@wustl.edu; 3 Department of Microbiology and Immunology, Tulane University School of Medicine, New Orleans, LA 70118, USA; tuan_phan@hhv-6foundation.org; 4 HHV-6 Foundation, Santa Barbara, CA 93108, USA; 5 Institute of Virology, Freie Universität Berlin, 14163 Berlin, Germany; cosimazimmermann@hotmail.com; 6 Division of Pulmonary and Critical Care Medicine, Department. of Internal Medicine, University of Michigan Medical School, Ann Arbor, MI 48109, USA

**Keywords:** herpesviruses, roseoloviruses, human herpesvirus 6, human herpesvirus 7, murine roseolovirus, porcine cytomegalovirus, Alzheimer’s disease, xenotransplantation

## Abstract

Viruses of the genus *Roseolovirus* belong to the subfamily *Betaherpesvirinae*, family *Herpesviridae.* Roseoloviruses have been studied in humans, mice and pigs, but they are likely also present in other species. This is the first comparative analysis of roseoloviruses in humans and animals. The human roseoloviruses human herpesvirus 6A (HHV-6A), 6B (HHV-6B), and 7 (HHV-7) are relatively well characterized. In contrast, little is known about the murine roseolovirus (MRV), also known as murine thymic virus (MTV) or murine thymic lymphotrophic virus (MTLV), and the porcine roseolovirus (PRV), initially incorrectly named porcine cytomegalovirus (PCMV). Human roseoloviruses have gained attention because they can cause severe diseases including encephalitis in immunocompromised transplant and AIDS patients and febrile seizures in infants. They have been linked to a number of neurological diseases in the immunocompetent including multiple sclerosis (MS) and Alzheimer’s. However, to prove the causality in the latter disease associations is challenging due to the high prevalence of these viruses in the human population. PCMV/PRV has attracted attention because it may be transmitted and pose a risk in xenotransplantation, e.g., the transplantation of pig organs into humans. Most importantly, all roseoloviruses are immunosuppressive, the humoral and cellular immune responses against these viruses are not well studied and vaccines as well as effective antivirals are not available.

## 1. Introduction: Herpesviruses

Herpesviruses are large DNA viruses with a genome of 120–230 kbp in size. Their genome is enclosed by an icosahedral nucleocapsid, which is surrounded by the tegument and a lipid envelope containing viral glycoproteins [1]. The linear double-stranded (ds) DNA encodes the so-called core genes that form seven gene clusters and are conserved among herpesviruses. Additional accessory genes are often virus-specific and contribute to the cell tropism, manipulation of cellular processes, evasion of host immune system and latency [2]. Nine human herpesviruses have been discovered that cause various clinical disease and are classified into the alpha-, beta- and gammaherpesvirinae. Roseoloviruses belong to the *Betaherpesvirinae*: roseoloviruses that infect humans, swine, and mice and have a high prevalence in their respective hosts and will be addressed in this review.

## 2. Human Roseoloviruses: HHV-6A, HHV-6B, and HHV-7

Three human roseoloviruses have been identified, human herpesvirus 6A (HHV-6A), 6B (HHV-6B), and 7 (HHV-7). HHV-6A was first isolated from PBMCs of patients with lymphoproliferative disorders by Salahuddin, Ablashi and colleagues in 1986 and was initially named the human B-lymphotropic virus [3]. HHV-6B was described by Lopez and colleagues two years later [4]. Based on genetic, immunological, and biological differences of virus isolates [5,6], HHV-6A and HHV-6B were finally classified as two separate species by the International Committee on Taxonomy of Viruses [7]. HHV-7 was first isolated in 1990 from T lymphocytes of a healthy blood donor [8]. In this part of the review, we will address the general structure and life cycle of the human roseoloviruses, as well as their biological and pathological significance.

### 2.1. Genomic Organisation and Replication of HHV-6A/B

HHV-6A/B virions are approximately 200 nm in diameter [9,10] and contain a dsDNA genome of about 160–162 kbp [11]. The genome contains a unique region (U) that is flanked by terminal direct repeats (DR) of 8–9 kb at each end. Both DNA strands contain coding sequences. Differences in gene sequences, splicing, and the resulting difference in the gene expression profiles between HHV-6A and -6B are likely responsible for the distinct biological properties of the two viruses [12,13].

For HHV-6A/B, 119 open reading frames (ORF) have been predicted, 9 of which are different between HHV-6A and -6B [11,12]. The genomes of HHV-6A and -6B are co-linear and have a 90% nucleotide identity [14]. The highest sequence divergence is observed in the immediate-early 1 (IE-1) region, the genome ends (U86 to DR_R_, excluding U94), and the glycoprotein gp82/105 (U100) [15,16]. Unique to the HHV-6A/B genome, among others, are the chemokine encoding gene U83 [17] and U94, which has a high sequence identity of 97.6% between HHV-6A and -6B [18]. 

HHV-6A/B preferentially infects activated CD4^+^ T-lymphocytes [19,20]. Other cell types are also permissive to infection in vivo or in vitro, although productive replication tends to be limited [21]. This suggests that cellular restriction factors act beyond entry [22], as this is the case for the cellular nuclear domain 10 (ND10) complex, which antagonizes viral transcription [23]. This broad tropism of HHV-6A/B was initially attributed to the complement regulatory receptor CD46 [24], which is ubiquitously expressed on all nucleated cells [25,26] and is described to facilitate entry of HHV-6A and certain HHV-6B strains [22,27]. Intriguingly, several isoforms of CD46 have been identified that differ in their ability to allow HHV-6B infection [27]. Tang and colleagues [28] identified an alternative receptor that is specific for HHV-6B, CD134, a member of the tumor necrosis factor receptor (TNFR) superfamily and a marker mainly expressed on activated CD4^+^ T cells [29]. However, HHV-6B can infect a wide range of target cells suggesting that it can utilize a yet unknown cell receptor [28]. In research, the T-cell lines Jjhan and HSB-2 are mainly used for HHV-6A, while MT4 and Molt-3 T cells allow HHV-6B propagation [6,30,31]. Both HHV-6A/B can also can be propagated in SupT1 cells [32]. 

HHV-6A/B encodes eight viral glycoproteins. gH, gL, gM, gN, gB are conserved among herpesviruses, while gO is conserved among betaherpesviruses, and gQ1 and gQ2 are specific to roseoloviruses [33,34]. HHV-6A uses the gH/gL/gQ1/gQ2 complex to bind the cellular receptor CD46 [35], with gQ1 and gQ2 playing a key role in this interaction [36,37]. In the case of HHV-6B, gQ1 and gQ2 are necessary and sufficient for CD134 binding [38]. Upon binding to the receptor, HHV-6A/B enter the cell via endocytosis [39]. In the endosomal compartment, the viral envelope fuses with the endosomal membrane and releases the capsid into the cytoplasm. The capsid is then transported along microtubules to the nucleus. The viral DNA is subsequently released through the nuclear pore complex into the nucleus. In the nucleus, the viral DNA circularizes and gene expression is initiated in a coordinated manner: immediate-early (IE) genes are expressed first and activate the early (E) genes implicated in replication, metabolism and blockage of antigen processing [40]. The late (L) genes are dependent on the synthesis of viral DNA and encode structural proteins for the capsid, teguments, and envelope [1,41]. The HHV-6A/B replication cycle takes approximately 72 hrs and is initiated by binding of the origin binding protein (OBP, U73) to the origin of lytic replication (ori-Lyt) located between U41 and U42 [1,18]. In the nucleus, OBP binding to the oriLyt initiates a partial denaturation of the DNA [42,43,44]. For DNA replication, a set of conserved virus-encoded proteins are required for efficient virus replication: the heterotrimeric helicase–primase complex (U43, U74 and U77), the single-strand (ss) binding protein (U41) that protects the ssDNA template [2,45], the DNA polymerase U38 that exhibits 3’–5’ proofreading exonuclease and RNaseH activity [46], and the DNA polymerase processivity factor U27 [47].

Amplification of HHV-6A/B DNA via rolling circle replication results in the formation of linked head-to-tail concatemers. These concatemers are then cleaved at the cleavage/packaging signals pac1 and pac2 at the ends of the DRs, allowing the encapsidation of unit-length virus genomes [2,45,48]. Differences in oriLyt and pac sites have been described between the two variants HHV-6A and -6B [49]. Next, the capsid buds into the perinuclear cisternae and acquires a first envelope. The nucleocapsid is then released into the cytoplasm by a de-envelopment step, where it acquires the final teguments. It subsequently buds into the trans-Golgi network, where it acquires its final envelope with glycoproteins [50]. Finally, the mature particle is released from the cell by exocytosis or cell lysis [2,51]. In contrast to most other herpesviruses, viral glycoproteins of HHV-6A/B are not detectable at the cell surface of infected cells [52,53].

### 2.2. Latency and Integration of HHV-6A/B

A hallmark of herpesviruses is that they establish a persistent infection in the host for life, termed latency. While most human herpesviruses maintain their genomes as extrachromosomal circular episomes during latency [41], HHV-6A/B integrate their genome into the telomeres of latently infected cells. Luppi and colleagues provided the first evidence that the HHV-6 genome was linked to high molecular weight cellular DNA [54]. Further studies confirmed that no linear viral genomes or free circular episomes are present and that HHV-6A/B integrates into the telomere region of cellular chromosomes [55,56]. The HHV-6A/B genomes harbor telomeric repeat arrays identical to the host telomeres at their ends that facilitate this integration process [57,58,59]. Sequencing analysis confirmed that the right DR region (DR_R_) of the viral genome is fused to the end of the chromosome, while the pac1 and pac2 sequences are lost during the integration process. The viral TMRs on the other end of the viral genome are likely extended to restore the telomeres [60]. It has been proposed that the HHV-6A/B encoded putative integrase U94, a homologue of the human adeno-associated virus type 2 (AAV-2) rep gene [61,62], facilitates HHV-6A/B integration and aids in the establishment latency [63]. However, Wallaschek et al. recently demonstrated that U94 is dispensable for HHV-6A integration into telomeres [64]. Several models for HHV-6A/B integration have been proposed including homologues recombination pathways like the DNA repair mechanisms break-induced replication (BIR) or single-stranded annealing (SSA) (reviewed in [57,65]). However, the important cellular recombinase (Rad51) and two highly conserved viral recombination proteins (U41 and U70) that also facilitate SSA are dispensable for integration [66]. So far, the underlying mechanisms of viral integration remains poorly understood. Although previous studies have observed HHV-6A/B reactivation inducing diseases in patients [67,68,69,70], reactivation of the virus genome in vitro is challenging. In most cell lines, induction of reactivation using various reagents only results in the expression of some viral genes without the production of viral particles [55,71,72]. Analysis of the chromatin profile of the integrated HHV-6A genome revealed that the viral DNA was transcriptionally silent, highly condensed, and associated with repressive histone modifications (H3K9me3 and H3K27me3) [73], suggesting that epigenetic factors are involved in the establishment and maintenance of latency. Similarly, Peddu and colleagues assessed gene expression signatures in various tissues and detected low levels of HHV-6A and -B gene expression across several tissues, with the highest levels present in the brain in the case of HHV-6A [74].

### 2.3. Genome, Replication, and Latency of HHV-7

The HHV-7 genome is about 145–153 kbp in size [75,76], and its unique region is also flanked by 6–10 kpb direct repeat sequences, and the whole genome comprises 84 ORFs [76] with one HHV-7 specific gene (U55B) and no homologue of U94 [14,18]. The cellular CD4 protein has been identified as one receptor used by HHV-7 for infection [77]. Replication of HHV-7 is thought to occur comparably to HHV-6A/B; however, very few studies have investigated HHV-7 replication. So far, only one report proposes chromosomal and germline integration of HHV-7 [78]; however, further work needs to be done to confirm HHV-7 genome integration.

### 2.4. Prevalence and Transmission of HHV-6A/B

HHV-6B is a ubiquitous virus that infects >90% of people within the first two years of life [79], while HHV-6A is less prevalent in humans. HHV-6B is transmitted via the saliva, and primary infection occurs in early childhood [79,80,81]. The route of transmission for HHV-6A remains unknown. Beyond that, HHV-6A/B can also be transmitted vertically through the integration of the virus genome into germ cells, resulting in offspring that harbors the identical integrated virus in every cell of the host. This condition is transmitted in a Mendelian manner and is termed inherited chromosomally integrated HHV-6 (iciHHV-6). Transmission of iciHHV-6 from parents to offspring was first described by Daibata et al. in 1998 [82]. It is present in approximately 1% of the human population [83], possibly corresponding to as many as 75 million people. In addition, iciHHV-6A/B positive mothers can also reactivate the virus and transmit it to their offspring via the placenta [70,84,85], despite low levels of virus shedding in the genital tract of pregnant or nonpregnant women [86]. 

### 2.5. Prevalence and Transmission of HHV-7

HHV-7 infection is also acquired during childhood [87] and, thus, is also widespread in the human population [88]. In healthy individuals, HHV-7 is present in the saliva [89] suggesting that smear or droplet infection contributes to transmission. In contrast to HHV-6A/B, a large study of cord blood showed that congenital infection is unlikely for HHV-7 [90]. Reactivation of HHV-7 after transplantation has been reported; however, no HHV-7-specific clinical symptoms were observed [91].

### 2.6. Clinical Consequences of HHV-6A/B

Primary infection with HHV-6B and HHV-7 is best known for causing roseola (exanthema subitum, roseola infantum) in immunocompetent patients. Roseola is the most common cause of childhood rash and accounts for a significant portion of childhood hospitalizations. The prevalence of roseola peaks between 7 to 13 months of age and classically begins with a fever of 3 to 5 days that may exceed 40 °C (104 °F) followed by a blanching macular or maculopapular rash starting at the neck and trunk that can spread to the face and extremities [92]. Roseola is almost always diagnosed clinically, and treatment is supportive as roseola is a self-limited disease. However, complications of roseola can include febrile seizures with some patients experiencing febrile status epilepticus [93]. A large study of nearly 200 infants found that 32% of febrile status epilepticus cases were associated with active HHV-6B infection, and 7% also had active HHV-7 infection [94].

Most cases of HHV-6A/B infections that require antiviral treatment occur in the transplantation setting, in which patients are usually immunocompromised. The endogenous reactivation of the latent virus, in combination with immunosuppressive therapies, accounts for most of these symptomatic infections. Primary infections can also occur in pediatric transplant patients and negatively impact the outcome of liver transplantations [95]. HHV-6 reactivation occurs in about 44% (range 13.9–93.6%) of allogeneic hematopoietic cell transplant (alloHCT) recipients [68] and 4.3% to 54.5% of liver transplant recipients [95]. The reason for the great variability in reactivation rates is due to varying levels of immunosuppression and antiviral prophylaxis. In addition, differences in viral load thresholds, sampling (e.g., plasma vs. whole blood) and PCR methods (quantitative vs. qualitative) could account for this variability. The underlying indication for transplantation can also affect the frequency of HHV-6 A/B reactivation: for example, patients with biliary atresia have an increased risk of HHV-6 reactivation [96]. Similarly, patients who undergo liver transplantation for acquired HBV/HCV are more likely to have HHV-6A/B infections after liver transplantation [97], leading to higher fibrosis scores [95].

As HHV-6A/B are ubiquitous pathogens, characterizing relationships between the viruses and clinical disease, especially, has been difficult. A causal relationship after transplantation has been established for HHV-6A/B encephalitis [98] and hepatitis after transplantation [95,99,100]. Beyond that HHV-6A and B have been associated with a number of clinical syndromes including acute graft-versus-host disease (aGVHD) [68], post-hematopoietic cell transplantation (HCT) lower respiratory disease [101], idiopathic pneumonia syndrome (IPS) [102], gastrointestinal diseases (GI diseases) [95], myocarditis [103,104], female infertility [105,106], multiple sclerosis (MS) [107], drug-induced hypersensitivity syndrome/drug rash with eosinophilia and systemic symptoms (DIHS/DRESS) [108,109] and Hashimoto´s thyroiditis [110]; however, further research is required to characterize these relationships. Treatment of HHV-6-associated syndromes involve the use of antivirals (e.g., ganciclovir or foscarnet) and reduction of immunosuppression [111]. Prophylaxis and pre-emptive antiviral therapies for HHV-6-associated syndromes are currently not recommended for either HCT recipients [112] or liver transplant recipients [95]. 

Two recent studies indicated that HHV-6A and HHV-7 play a role in Alzheimer’s disease (AD). In the first study, a multiscale analysis of independent Alzheimer’s disease cohorts identified transcriptomic HHV-6A and HHV-7 signatures that were enriched as a function of AD progression [113]. Increased HHV-6A and HHV-7 DNA and RNA were identified in brains of subjects with AD, and these findings were replicated in two additional, independent cohorts. Furthermore, these investigators observed that HHV-6A not only mediates the impact of the APOE4 isotype on AD risk, but also modulates the expression of key regulators (*PSEN1, PSEN2, BACE1, APBB2,* and *CLU*) of amyloid precursor protein (APP) processing. These findings were complemented by a separate study showing that Aβ42 oligomers bind and agglutinate HSV1 and HHV-6, with the viral envelope glycoprotein (gB) as the most likely binding target [114]. These data suggest that rapid seeding of β-amyloid in the brain serve as an innate mediator that protects against herpesvirus infections; however, these protective measures accelerate β-amyloid deposition and potentially result in the progression of AD. These studies provide a potential mechanism of herpesvirus-induced Aβ-amyloidosis in AD patients that should be further investigated in future studies. 

### 2.7. Clinical Consequences of iciHHV-6A/B 

The clinical significance of iciHHV-6 is just beginning to be elucidated. In a recently published study of 4,319 HCT donor-recipient pairs (*n* = 8,638) who were screened for iciHHV-6, investigators identified 60 HCT recipients (1.4%) and 40 donors (0.9%) with iciHHV-6 [115]. In the case of 13 HCTs, both donor and recipients were iciHHV-6 positive, resulting in 87 HCTs (2%) in which the recipient, donor, or both harbored iciHHV-6. HCMV viremia was more frequent among recipients with iciHHV-6. Patients with iciHHV-6 were almost twice as likely to develop grade II–IV aGVHD compared to those without iciHHV-6 [115]; a separate meta-analysis of over 3000 alloHCT patients suggested that HHV-6 reactivation (in the absence of iciHHV-6) was also associated with a nearly 3-fold risk of developing grade II–IV aGVHD [68]. 

Some studies have suggested that histone deacetylase (HDAC) inhibitors [68] and steroids [55,116] can trigger iciHHV-6 reactivation, and case reports have shown that iciHHV-6 can be horizontally transmitted via liver transplantation and subsequently reactivated causing fatal clinical disease [117]. iciHHV-6 is currently diagnosed via whole blood qPCR (>log 5.5 copies per mL) or digital droplet PCR (ddPCR), nail clippings, or hair follicles [83]. There are currently no guidelines for the treatment of iciHHV-6 disease; clinical judgment should be exercised when treating iciHHV-6^+^ patients.

### 2.8. Clinical Consequences of HHV-7 Infections

Aside from roseola, the role of HHV-7 in the clinical setting has yet to be defined. Similar to HHV-6A/B, HHV-7 replicates in CD4^+^ lymphocytes, it, however, does not appear to be as pathogenic as HHV-6 A/B. HHV-7 infection is almost always asymptomatic, although it seems to predispose kidney transplant recipients to HCMV infections [118,119]. Isolated cases of encephalitis [120], Guillain–Barre syndrome [121], acute myelitis [122], optic neuritis, and meningitis [123] have been described. HHV-6A and HHV-7 infections have also recently been tied to Alzheimer’s disease, which was discussed in Section 2.6. Diagnostic assays for HHV-7 are used primarily in research settings, and there are currently no guidelines for treating HHV-7 infections.

### 2.9. Immune Response to HHV-6A/B and HHV-7 Infections

A number of studies investigated the response to HHV-6A/B; however, little is known about the responses to HHV-7 infections. There are a number of challenges in studying immune responses against these viruses. One is the high seroprevalence of these viruses, which makes it hard to find individuals that serve as negative controls. In addition, the frequency of specific memory B and T cells is very low in latently infected humans, the virus genome is complex, and we lack animal models to assess immune responses [124,125]. 

Maternally derived antibody titers dwindle over 3–9 months after birth making the children susceptible to infection [125]. Upon primary infection with HHV-6B, lifelong immune responses are induced. Like other herpesviruses, HHV-6A/B can evade CD8^+^ T cells by downregulating MHC I, which may account for the low frequency of virus-specific CD8^+^ T cells. Compared to other herpesviruses, HHV-6A/B specific cell-mediated response is delayed in primary infection [124], which could be attributed to its strong tropism for lymphocytes and their depletion [126]. This depletion of CD4^+^ T cells has been previously shown for HHV-6B in patients [127,128,129]. In a humanized mouse model of HHV-6 infection, both HHV-6A and HHV-6B were able to efficiently infect human thymic tissue implanted in SCID-hu Thy/Liv mice, leading to graft destruction [130]. HHV-6A was associated with CD4^+^/CD8^+^, CD4^+^/CD8^−^, and CD4^−^/CD8^+^ thymocyte depletion, whereas HHV-6B selectively depleted only CD4^+^/CD8^+^ and CD4^+^/CD8^−^ thymocytes.

HHV-6B infection of T cells can suppress CD4^+^ responses [131] by inducing apoptosis, inhibiting IL-2 synthesis, arresting the cell cycle, and down-modulating T-cell receptors and MHC-I [125]. HHV-6A-specific CD4^+^ T-cells can produce IFN-γ and degranulate when presented with the whole virus or peptide antigen, suggesting HHV-6A-specific cytotoxicity [124,132]. In pediatric HCT patients, increased proportions of perforin-expressing CD8^+^ T cells have been temporally associated with HHV-6 clearance [124,133]. HHV-6A can also infect natural killer (NK) cells leading to the de novo expression of CD4, which predisposes these NK cells to HIV infection [134]; however, NK cells have enhanced killing potential against HHV-6A-infected cells through a mechanism mediated by IL-15, which triggers IFN-γ from both CD4^+^ and NK cells [135]. On the other hand, HHV-6B infection of T cells results in the down-regulation of ligands that normally would activate NK cells, allowing HHV-6B to evade NK cell attack [136]. Notably, while HHV-6A can efficiently infect cytotoxic effector cells like CD8^+^ T cells and NK cells, HHV-6B does not productively infect these cells [137] and preferentially infects CD4^+^ T cells.

Primary HHV-6B infection is typically a mild febrile disease, and most children recover rapidly without complications. In a study of cell-mediated immune response to HHV-6 (U54) in healthy children without any underlying immune disorder or infectious disease, the amount of IFN-γ, IL-2, and TNF-α-secreting T cells were measured by flow cytometry after 10 days of pre-sensitization and 6 hours of re-stimulation with mixtures of pooled overlapping peptides from U54 [138]. All individuals showed a virus-specific response for at least one cytokine in either CD4^+^ or CD8^+^ cells and the percentage of HHV-6-specific TNF-α response in CD4^+^ (48%) and CD8^+^ (56%) were always the highest. Higher frequencies of HHV-6-specific TNF-α producing CD8^+^ T cells are correlated with increased age, possibly reflecting repeated stimulation by viral persistence and subclinical reactivation. There is little research on the immune response of HHV-7 in vivo, though HHV-7 is more cell-associated, less cytopathic, and grows with slower kinetics in vitro than HHV-6 [139].

HHV-6B reactivation after allogeneic stem cell transplantation causes delayed long-term T-cell reconstitution, which is the thymus-dependent phase of T-cell reconstitution after HCT [140,141]. This could contribute to the increased risk of developing grade II–IV aGVHD [68,137]. aGVHD is treated with steroids, which has been shown to increase the risk of HHV-6 reactivation in both HCT and solid organ transplant patients [137]. Given that HHV-6B reactivation is associated with steroid therapy and grade II–IV aGVHD, it is not surprising that some steroid-refractory cases of aGVHD are associated with increased detection of HHV-6B infection [142,143]. Further studies designed to address the role of HHV-6B in steroid-refractory aGVHD and whether antiviral treatment can prevent morbidity and mortality in alloHCT recipients are needed. 

Currently, a key area of research is the development of HHV-6-targeted immunotherapies. Prominent antigens targeted by the antibody response include major antigenic virion protein U11, glycoprotein gH (U48), and gQ (U100), polymerase processivity factor (U27), latent antigen U94, and tail-anchored membrane protein U24 [125]. Monoclonal neutralizing antibodies targeting gH, gQ, and gB have been described [144,145,146,147,148]. A phase I clinical trial showed that the adoptive transfer of peptide-expanded T cells for HHV-6, HCMV, BKV, EBV, and AdV was safe, did not induce high levels of cytokines and did not induce allo-specific responses [125,149]. Expansion of HHV-6B T cells was performed with overlapping peptides of the immediate early protein 1 (U90) and the tegument proteins U11 and U14. The transferred population was ~60% CD4+ and 35% CD8+ T cells; 30% of the developed T cell lines had responses to all 5 viruses, and 70% had responses to ≥3 viruses. Specifically, the expanded T cell population was able to produce multiple effector cytokines and kill both peptide-loaded and HHV-6B wild-type virus-infected target cells. The investigators concluded that adoptive T-cell immunotherapy for HHV-6 represented a practical approach for controlling HHV-6 infections in transplant patients. 

## 3. Murine Roselovirus: MTV/MRV

Murine roseolovirus (MRV) was first discovered by Rowe and Capps from laboratory mouse colonies and wild mice in 1961 [150]. It was named mouse thymic virus (MTV) based on the distinctive pathology of massive necrosis and atrophy of the thymus in infected neonates [150]. The morphology of MTV on electron microscopy suggested that it was a herpesvirus [151]. This virus was designated as murid herpesvirus 3 by the International Committee on Taxonomy of Viruses (ICTV). Due to its suspected T lymphotropic property, it has also been referred to as mouse T lymphotropic virus (MTLV) [152,153]. 

A viral stock with similar properties of MTV infection was sequenced in 2017 by Patel et al. and revealed that it is a herpesvirus closely related to the human roseoloviruses, and thus was named murine roseolovirus (MRV) [154]. Because there was no clear passage history of the MRV stock, its relationship with MTV was not immediately determined [154,155]. The complete genome of a stock of MTV kept at Charles Rivers Laboratory was later sequenced in 2018 (GenBank: MG595964.1) [156], and demonstrated >99% sequence homology to MRV. This provides evidence that MRV and MTV are likely the same virus. While it remains unclear how many passages separate the two stocks, the nearly identical sequence homology suggests that the viral genome maintains a high degree of fidelity over time and passages. For the sake of clarity, we will use the name MRV throughout this review of roseoloviruses.

### 3.1. Genomic Organization and Latency 

The MRV genome is 173,861 bp and is comprised of a unique region flanked by a 6476 bp direct repeat (DR) [154]. In silico analysis predicted 128 open reading frames (ORFs). ORFs 1–124 occur within the unique region, while ORFs 125–128 are within the DR region. The MRV genome contains homologous genes in each of the herpesvirus core gene categories and contains homologs to the beta-herpesvirus signature genes. Ninety-seven ORFs have homologues in other herpesviruses, and 65 ORFs are highly homologous HHV6 or HHV7 genes. Interestingly, the MRV genome is missing homologs to human roseolovirus genes, U48A, U80.5, and U99. Additionally, there is no clear homolog to the immediate-early 1 (IE1) gene, U90. ORF107 is a 381 bp gene that has the closest homology to U90. By comparing the MRV DNA polymerase (ORF 51), glycoprotein B (ORF 53), and viral ssDNA binding protein (ORF 55) with the corresponding proteins of human beta herpesviruses, it has been found that the phylogenetical distance from MRV to HHV-6 is similar to that from MRV to HHV-7 [154].

Little is known about MRV latency, but it is likely that it has a latent phase similar to all other known herpesviruses. Evidence for latency comes from observations that infection could be transmitted via salivary gland isolated as many as 241 days after infection, despite a low viral load [150]. The MRV genome does not contain hexameric repeats thought to be necessary for integration, although integration has yet to be studied [157].

### 3.2. Prevalence and Transmission

MRV is a natural pathogen of mice with high prevalence in wild mice. In a study conducted in the northwest UK, 78% of newly caught wild mice were serologically positive for MRV [158]. The transmission of MRV is through direct contact as well as from lactating mothers to suckling mice [159] through the transmammary passage [152]. The contact transmission is likely mediated by saliva, as MRV infection is persistent in the salivary glands, and the lytic virus is continuously shed for months after infection [160]. The viral transmission among mouse cage-mates requires prolonged contact with each other [159]. Not surprisingly, mice housed in the same room but different cages with infected mice do not get infected [160]. Vertical transmission by the transplacental route was not detected in fetuses of laboratory mice [159]. Since the neonates that acquired MRV from their mothers do not develop antibodies against MRV [159], serological tests may underestimate the prevalence of MRV infection in wild mouse populations.

### 3.3. Pathogenicity

Wild mice with positive serology appear to be asymptomatic [158]. Experimental inoculation of MRV into neonates within the first four days after birth causes thymus necrosis and failure to gain weight [150,160]. However, neonates older than four days or adult mice do not show thymus necrosis after infection with MRV [150,160]; rather, a chronic infection of the salivary glands is established [160]. No virus was isolated from other organs of adult mice inoculated with MRV [160]. Mice that acquired MRV infection via direct contact with cage-mates or via lactating mothers do not develop thymus necrosis [159].

Macroscopic necrosis of the thymus is visible in neonates at seven days post-inoculation (dpi) and reaches a maximum between days 10 and 14 [160]. Transient necrosis of the lymph nodes and spleen also occur in MRV inoculated neonatal mice, which starts at day 7 and peaks at 14 dpi [161]. In these infected lymphoid organs, lymphocytes, epithelial reticular cells, macrophages, and thymic nurse cells contain viral particles and filamentous structures in both the nucleus and the cytoplasm [162,163]. By three weeks post-inoculation (wpi), the histology of the infected thymus becomes normal again [160], and the total number of cells in the mesenteric lymph node is recovered to the levels of normal mice [161]. The infected neonates also transiently fail to gain weight, but by 3 wpi, their body weight becomes similar to control mice [160]. By 6 wpi, the weight of the infected thymus is comparable to normal controls [150,160]. Interestingly, although both CD4^+^ and CD8^+^ cells get infected in the thymus and spleen, only the CD4^+^ CD8^+^ and CD4^+^ CD8^−^ lymphocytes are sensitive to apoptosis [162,163]. The loss of T lymphocytes apparently causes transient host immune suppression. For example, rejection of unmatched skin grafts is delayed upon infection with MRV [161]. 

MRV can also cause autoimmune disease after acute infection and with a long-term impact. Some strains of mice, such as BALB/c, develop autoimmune gastritis three months after neonatal infection with MRV [164]. The mice that develop gastritis have high titers of anti-parietal cell autoantibodies. This autoimmune disease appears to be CD4^+^ T cell-mediated. Adoptive transfer of CD4^+^ T cells from adult mice that had been neonatally infected with MRV to uninfected nude mice results in the development of autoimmune gastritis [164]. Conversely, adoptive transfer of normal CD4^+^ T cells, but not CD8^+^ T cells, from uninfected syngeneic adult mice can prevent autoimmune gastritis in mice that were neonatally infected with MRV [164]. HHV-6 may also induce autoimmunity. HHV-6A mRNA and DNA is highly prevalent in biopsies of patients with autoimmune thyroiditis compared to controls [110], and HHV-6A was found to alter the expression of several microRNAs in a pattern that is considered a marker for patients with autoimmune thyroid disease [105].

Like its human herpesvirus homologs HHV-6 and HHV-7, MRV can reactivate from latency after HCT. In minor histocompatibility antigen mismatched alloHCT recipients (i.e., bone marrow cells of B10.D2 mice → BALB/c neonatally infected adult mice), replicating MRV can be detected in the lung, liver, skin, and gut at two weeks after transplantation [102]. The reactivation of MRV stimulates the infiltration of immune cells and is associated with weight loss, lung injuries and increased signs of graft-versus-host disease [102].

### 3.4. Immune Response and Antiviral Drugs

As discussed above, neonatal infection has a significant impact on the immune system, especially the T cell compartment, and results in a transient reduction in response to mitogen and graft-versus-host response [161,165,166]. While MRV appears to have a significant effect on CD4^+^ T cells, CD8^+^ T cells are important for controlling MRV infection. After viral infection, there is an increase in activated/effector CD8^+^ T cells, as demonstrated by increased expression of activation markers CD44 and CD69, and effector molecules granzyme B and interferon gamma [163]. This appears to be a polyclonal and antigen restricted response [163]. Supporting an important role of CD8^+^ T cells in controlling MRV infection, CD8 knockout mice are unable to recover CD4^+^ T cell levels and have an 80% mortality after neonatal infection [163]. Of note, this demonstrates that CD4^+^ T cell depletion occurs independently of CD8^+^ T cells. CD8 knockout mice also develop higher viral load compared to wild type mice [163]. TCR knockout mice demonstrate a similar phenotype [163].

Somewhat surprisingly, Rag knockout mice, in which T cell development is arrested in the DN3 stage, developed increased viral DNA levels in the spleen but not in the thymus compared to wild type mice [163]. This may suggest that while CD8^+^ T cells are important for controlling MRV infection, CD4^+^ T cell may be important for viral replication. This idea is supported by the finding that nude mice, which have a mutation in FOXN1 and lack a thymus or mature T cells, have decreased viral loads compared to wild type mice [167]. The ability of Rag knockout mice and nude mice to control MRV viral loads relatively well after neonatal infection might also suggest an important role of the innate immune system, although this has yet to be studied in depth.

B cells do not appear to play a significant role in controlling acute infection in neonates. Antiviral antibodies are not produced by neonates after infection but are produced in MRV infected adult mice [160]. Despite this, µMT mice, which lack mature B cells, do not demonstrate increased viral loads and recover CD4^+^ T cell levels similar to wild type mice [163]. Neonatal MRV infection does not seem to have a long-term impact on B cell response, as neonatal MRV infection do not alter the response to type III polysaccharide antibody levels as early as 1 wpi [168]. While an antibody response to MRV is maintained for as many as 152 dpi [160,169], the virus continues to be shed in saliva [170,171]. Taken together, this suggests that B cells and antibody production do not play a significant role in controlling acute infection.

An important consideration in understanding the immune response to MRV is that most of the studies have focused on neonatal infection. The infection of adult mice does not produce thymic atrophy or weight loss. Additionally, we have found that adult mice do not develop CD4^+^ T cell depletion (Patel, Bigley, et al., unpublished observation). The establishment of MRV as a homolog to human roseolovirus provides an in vivo system to study how the immune system controls infection as well as the short-term and long-term impact of these ubiquitous viruses on the immune system.

## 4. Porcine Roseolovirus: PCMV

### 4.1. Genomic Organization and Latency

The name porcine cytomegalovirus (PCMV) is misleading, as the virus is a roseolovirus closely related to HHV-6, HHV-7, and MRV. On the genetic level, it is quite distinct from the human cytomegalovirus (HCMV or HHV-5). In order to clarify this, the virus will be called PCMV/PRV (porcine roseolovirus) in this manuscript until the classification as suid herpesvirus (suid betaherpesvirus 2, or suid herpesvirus 2, SuHV2) becomes common.

The virus genome is 128,367 bp and, therefore, is shorter than HHV-6A/B and HHV-7 [172]. Like in all roseoloviruses, the genome contains a unique region (U) flanked by direct repeats (DR). The 363 bp DR of PCMV/PRV is also drastically shorter compared to those of other roseloviruses. In contrast to human roseoloviruses, no telomeric repeat arrays were identified at the ends of the PCMV/PRV genome. Seventy-nine open reading frames were identified in the unique region, and 69 of them have homologs in HHV-6 and HHV-7. Among the conserved genes are the immediate-early genes, genes associated with replication, nucleotide metabolism and DNA repair. Other genes encode the glycoproteins of the envelope, capsid, tegumemt, and virus assembly proteins. The U39, U46, U48, U72 and U82 have homologs in the other roseoloviruses and encode for the glycoproteins B (gB), N (gN), H (gH), M (gM) and L (gL), respectively, which play a role in virus entry and are required for growth and maturation. The products of PCMV/PRV U54A and U54B are two pp65 proteins with vast differences to their homologs in the other roseoloviruses.

Like all herpesviruses, PCMV/PRV establishes latency [173] and can be reactivated upon immunosuppression, antigenic stimulation, pregnancy, and other stimuli. Since the lifetime of farm pigs is very limited, detailed studies on latency have not been performed yet. It remains unknown how the virus maintains its genome during latency, as it does not harbor telomeric repeats that facilitate the integration of HHV-6A/B into host telomeres. In the absence of viral telomeres, PCMV/PRV may use the integrase of porcine endogenous retroviruses (PERVs), which are in contrast to human endogenous retroviruses (HERVs) in human tissues, massively expressed in pig tissues [174]. 

### 4.2. Prevalence and Transmission

PCMV/PRV is present throughout the world, and over 90% of herds have been exposed to infection. PCMV/PRV is found in tissues throughout the body; it is excreted in discharges from the nose and eyes, urine, and farrowing fluids. It is also transmitted via the boar through semen and crosses the placenta to infect piglets before birth [173,175]. 

### 4.3. Pathogenicity

Most PCMV/PRV infections are sub-clinical. It causes rhinitis, pneumonia, anemia, and fever in newborn piglets. Rhinitis in newborn piglets can be severe enough to cause nasal haemorrhage. Gastrointestinal and neurological signs also have been reported [173,175]. Severe clinical signs are only seen if PCMV/PRV infects a sow for the first time during a late stage of pregnancy. Signs include fetal deaths, mummified fetuses, stillbirths, and weak piglets. The sow may develop a slight fever and become lethargic. However, since PCMV/PRV is an immunosuppressive virus, it may allow opportunistic bacterial and viral co-infections and enhance their pathogenicity. PCMV/PRV mainly inhibits the immune function of T lymphocytes and macrophages [176]. PCMV/PRV infection is usually associated with opportunistic bacterial infections due to immune modulation and suppression [175]. Based on the results of clinical testing, some PCMV/PRV-infected animals were co-infected with porcine reproductive and respiratory syndrome virus (PRRSV), transfusion transmitted virus (TTV1), and pseudorabies virus (PRV). In addition, *Streptococcus*, *Haemophilus parasuis, Actinobacillus,* and *Pasteurella suis* infections often occur following PCMV/PRV infection, making prevention and treatment more difficult [177]. A transcriptome analysis of PCMV/PRV -infected thymuses showed an up- and downregulation of immune-regulatory genes [178]. In this study, 2161 genes were up-regulated, among them IL-1α, IL-1β, IL-7, IL-8, and IL-16, and 3421 genes were down-regulated, among them IL-2, IL-12, TGFβ1 [178]. When porcine micro-RNAs (miRNA) were analyzed in PCMV/PRV-infected and non-infected porcine macrophages, the differentially expressed miRNAs are mainly involved in immune and metabolic processes [176]. Ninety-two, 107, 95, 77, and 111 miRNAs were significantly differentially expressed in lung, liver, spleen, kidney, and thymus after PCMV/PRV infection, providing insights into the regulatory mechanisms of miRNAs during infection by immunosuppressive viruses [179].

In vitro, it has been shown that allogenic stimulation [180], and mitogen-stimulation [181] of porcine peripheral blood mononuclear cells (PBMCs), as well as xenogenic immune responses upon transplantation of pig organs into baboons [182,183], are able to induce reactivation and enhance replication. These data suggest that immune responses against infections with other microorganisms can reactivate latent PCMV/PRV and may accelerate diseases induced by other viruses. 

### 4.4. Immune Response and Antiviral Drugs 

Virus-specific antibodies, including neutralizing antibodies, are found detected in infected pigs; however, these rather low responses are not sufficient to eradicate PCMV/PRV completely (for review see [173,175]). The immune responses allowed the establishment of immunological assays measuring anti-viral antibodies as indirect evidence of virus infection. Among these methods are a blocking ELISA using a part of the glycoprotein B (gB) of PCMV/PRV as an antigen [184] and a Western blot assay using the N- and C-terminal parts of gB [185]. These domains are highly conserved among the 46 published sequences of PCMV/PRV. Furthermore, the tegument proteins U54A and U54B of PCMV/PRV [172] were expressed, purified, and used as antigens in Western blot assays [186]. Using the recombinant gB protein of PCMV in an ELISA, the seroprevalence in Huang province, China was 96.4% [184]. A specific immunohistochemistry could be established detecting PCMV/PRV antigens in infected pigs [187] and baboons after orthotopic transplantation of PCMV-positive pig hearts [181] using a rabbit antibody against a Japanese PCMV/PRV isolate. Due to the high sequence similarity, antibodies against HHV-6 in humans have been shown to cross-react with the viral gB protein of PCMV/PRV [186].

In vitro replication experiments revealed that ganciclovir and cidofovir and, to a lesser extent, foscarnet and acyclovir inhibit PCMV/PRV replication [188]. However, in an in vivo study, ganciclovir was not very effective in inhibiting PCMV/PRV replication in baboons after transplantation of PCMV-positive pig organs [189].

### 4.5. PCMV/PRV and Xenotransplantation

The economic damage induced by PCMV/PRV in pig production is moderate, and therefore, there is no urgent need to eliminate the virus in pig farms. However, PCMV/PRV is of great importance in the context of xenotransplantation. Xenotransplantation using pig cells, tissues, and organs is considered the best solution to alleviate the shortage of human transplants to treat organ failure [190,191]. For numerous reasons, pigs are thought to be the best donor animal. Using multiple genetically modified pigs and improved immunosuppression regimens, remarkable survival times of pig islet cells for the treatment of diabetes and pig organs transplanted into non-human primates were achieved (for review see [190,191]).

Xenotransplantation using pigs may be associated with the transmission of potentially zoonotic porcine microorganisms. In addition to the hepatitis E virus, which is known to be transmitted from pigs and pig products to humans causing, under certain conditions, disease [192], PCMV/PRV is also a potential zoonotic virus [193]. PCMV/PRV was transmitted in pig to non-human primate xenotransplantation trials of kidneys and hearts and was the cause of a significant reduction of the survival time of the transplanted pig organs (for review see [194]). Similar results had been observed in two primate species (cynomolgus monkeys and baboons) [195,196], suggesting a devastating zoonotic effect may also be expected in humans. The mechanism of how PCMV/PRV causes the reduction of the survival time of the transplant is still unclear. It is also unknown whether PCMV/PRV can infect primate cells including human cells [181,197,198]. However, in baboon recipients of PCMV/PRV-positive hearts, PCMV/PRV-positive cells can be detected in all organs of the animal after transplantation, which could also represent disseminated pig cells expressing PCMV/PRV [181]. PCMV/PRV may influence the cytokine production, the coagulation system, and the endothelial cells, eventually leading to a multi-organ failure. Since there is no effective antiviral drug available [188,189] and no vaccine, prevention of transmission of the virus during xenotransplantation can only be successful by early weaning, colostrum deprivation, Caesarean section, and embryo transfer. PCMV-free pigs were also successfully obtained by early weaning [199,200,201], despite the observation that PCMV/PRV can also be transmitted via the placenta [175,202]. This will lay the foundation for a safe xenotransplantation in near future.

## 5. Comparative Analysis

Roseoloviruses are ubiquitous in nature, as HHV-6B, HHV-7, PCMV/PRV, and MRV have a seroprevalence between 75% and 95% in their respective host populations (Table 1). In addition, HHV-6B, MRV, and PCMV/PRV are easily transmitted mainly by saliva and other body fluids. When comparing the roseoloviruses in humans, pigs, and mice, it becomes obvious that the human roseoloviruses are better characterized than the porcine and murine counterparts (Table 1). 

Although all roseoloviruses are closely related and the general organization of the viral genome containing a unique region (U) that is flanked by direct repeats (DR) is identical, there are also some differences. MRV has the longest genome, PCMV/PRV the shortest (Table 1). MRV might have acquired more cellular genes in comparison to the other roseoloviruses. Consequently, the murine virus encodes 128 genes, the porcine virus only 79. Approximately 69 genes are common in all viruses and highly related. 

All roseoloviruses establish latency upon primary infection, although this property is not well studied in the case of PCMV/PRV and MRV. The human roseoloviruses harbor telomeric repeats at the end of their genome that facilitate integration of HHV-6A/B and possibly also HHV-7 into the telomeres of latently infected cells. This integration can also occur in germ cells resulting in individuals that harbor the virus in every cell of their body. MRV and PCMV/PRV do not harbor these telomeric repeats, raising the question of how their genomes are maintained during latency. 

Reactivation has been described for all roseoloviruses. The murine and human viruses can be reactivated by immunosuppression and upon transplantation of hematopoietic cells. Similarly, PCMV/PRV reactivation was also linked to immunosuppression in transplanted non-human primates. Reactivation of PCMV/PRV was also observed by allogenic and mitogenic stimulation of pig PBMCs [180,181]. 

All roseoloviruses suppress and evade aspects of immune function, a property characteristic for all herpesviruses, and many other viruses. The modulation of the human immune response by HHV-6 and HHV-7 is relatively well studied. Defined genes of human roseoloviruses have been shown to suppress type I IFN induction (HHV-6 IE-1protein), respond to CCL17, CCL19, CCL21, CCL22 (HHV-7 U12 and U51). HHV-6A encodes a β-chemokine (U83) that has chemotactic activity for CCR2, whereas the homologue in HHV-6B recruits CCR2, CCR4, CCR5, CCR6, and CCR8 positive cells (for review see [203]). The human roseoloviruses inhibit IL-2 gene expression (HHV-6B, but not HHV-6A U54), down-regulate CD3 expression (HHV-6A U24), expression of NK-activating ligands, and class I MHC molecules (HHV-7 U21) as well as induce apoptosis (HHV-6B U19) (for review see [203]). The genes and the mechanism inducing immunosuppression remain unknown in the case of PCMV/PRV and MRV. HHV-6A and HHV-6B infection can modulate the profile of cytokine and chemokine production (for review see [126]) and have been shown to reduce the production of IL-2 resulting in diminished immune cell proliferation. In PCMV-infected pig thymuses, gene expression of IL-1α, IL-1β, IL-7, IL-8, and IL-16 are up-regulated and IL-2, Il-12 and TGF β are down-regulated [178]. Roseoloviruses obviously interfere with cytokine and chemokine production to achieve successful immune evasion. A similar mechanism is also well known for other viruses, for example, retroviruses such as HIV-1 [204]. In HIV-1 infected individuals, the levels of IL-10 and IL-6 are significantly elevated. Changes in cytokine expression may also be the reason why the survival time of pig organs infected with PCMV/PRV in non-human primate recipients is significantly lower compared to that of organs from PCMV/PRV negative donor pigs [195,196].

A special mechanism of immune evasion by herpesviruses is their property to acquire cellular cytokines during virus evolution. Epstein–Barr virus, for example, encodes a viral analog to IL-10 [205], and HHV-6 encodes a chemokine [40]. Whereas other viruses, for example HIV-1, induce the cytokines indirectly to regulate the immune system [206], HHV-6 directly encodes an active chemokine as mentioned above (for review see [203]). Interestingly, it has been demonstrated that UV-treated HHV-6B is immunomodulatory in vivo, preventing the development of allergy by limiting the inflammation in the lungs of treated mice. This indicates that exposure to HHV-6B alters the immune response to subsequent challenges. One implication is that proteins in the virion have immunomodulatory properties that limit Th2-mediated inflammation. A direct immunosuppressive activity of a viral protein was also described in the case of retroviruses including HIV-1 [206] and measles viruses [207]. The transmembrane envelope protein of retroviruses induces modulation of cytokine gene expression [208]. Among the genes up-regulated by gp41 of HIV-1 were IL-6, IL-8, IL-10, but also MMP-1, TREM-1, and IL-1β. Most importantly, genes involved in innate immunity such as FCN1 and SEPP1 were found down-regulated [208].

Exposure of human macrophages to HHV-6A and HHV-6B impaired their ability to produce IL-12 upon stimulation with interferon gamma and lipopolysaccharide [209]. Since this effect was not abrogated by UV treatment of the viruses, this is another example of a direct influence of viral proteins on the immune system, in this case, possibly by interaction with the viral receptor CD46 [209]. Interestingly, the measles virus, which also uses CD46 as a receptor for cellular entry, also suppresses IL-12 production [210].

Roseolovirus infections are generally asymptomatic, likely reflecting the co-evolution of the natural host and the respective herpesviruses. The transient, self-limiting disease is observed in only some cases of roseolovirus infection, such as exanthema subitem and febrile seizures in children infected with HHV-6 and HHV-7. However, reactivation of roseolovirus during co-infection may alter disease caused by other microorganisms, possibly due to the immunosuppressive properties of roseoloviruses. For example, infection with HHV-6 increases the expression of CD4 on CD8^+^ cells resulting in an increased infection of CD8^+^ cells with HIV-1 [211,212]. Another mechanism of how HHV-6 contributes to HIV-1 pathogenesis is the enhanced death rate of co-infected CD4^+^ cells [213]. In contrast, the transmission of a roseolovirus into another species may have pathogenic consequences because the new species is not adapted to the virus. A significant reduction of the survival time of transplanted organs form PCMV/PRV-infected pigs into non-human primates is an example. Since it is still unclear but rather unlikely that PCMV/PRV infects primate cells, PCMV/PRV seems to modulate the cytokine production and endothelial cell function in the transplanted baboons (for review see [194,195,196]). Retroviruses, especially the immunodeficiency viruses, have also been shown to be asymptomatic after a long co-evolution. Whereas the simian immunodeficiency viruses (SIV) are apathogenic despite a high virus load in their natural hosts, trans-species transmission of SIV from chimpanzees resulted in highly pathogenic HIV-1 and HIV-2 in humans, or a trans-species transmission from sooty mangabeys to rhesus monkeys, resulted in highly pathogenic SIVmac [213].

The association of HHV-6 to neurological diseases, including multiple sclerosis (MS) and Alzheimer’s disease, is of great interest. However, the prevalence of human roseoloviruses is high, and thus, it is difficult to prove the causality of these diseases. It is still unclear whether the murine and the porcine models have potential for the study of Alzheimer’s disease. In the case of PCMV/PRV, neurological signs have been reported in infected pigs; detailed studies are still lacking [173,175]. In pigs, congenital PCMV/PRV infections have been associated with multifocal lesions distributed throughout the cerebrum and cerebellum, and the virus has been isolated from the central nervous system [175]. Now that PCMV/PRV free colonies have been established [199], it would be possible to study the effect of PCMV/PRV in the pig model. Furthermore, transgenic pigs being developed to study Alzheimer’s disease should be free of PCMV in order to obtain reliable results. MRV has been isolated from the brains of mice infected shortly after birth but not of those infected at the adult stage [160] No study on the impact of MRV on the brain were reported until now. Interestingly, infection with HHV-6A in mice transgenic for the human gene CD46 establishes a long-term persistence in the brain [214]; in human cells, it induces the expression of the envelope protein (Env) from the multiple sclerosis-associated retrovirus [215].

## 6. Roseoloviruses in Other Species

HHV-6A/B and HHV-7 in humans, PCMV/PRV in pigs, and MRV in mice are the best-studied roseoloviruses. However, roseoloviruses are likely present in many other species. When captive chimpanzees were tested using an HHV-6A/B immunofluorescence assay, 75–90% of the animals were found positive, depending on their habitat [216]. This prevalence is similar to the one of human roseoloviruses. Antibodies cross-reactive with HHV-6A/B were found in eight of 10 monkey species. These include cynomolgus monkeys, African green monkeys, chimpanzee, oran-utang, pig-tailed monkeys, siamang, Japanese monkey, and squirrel monkey, but antibodies were not detected in silvered lutongs or cotton-top tamarins. The prevalence of antibody was the highest in squirrel monkeys. These serological studies strongly suggested the existence of HHV-6-related viruses in different non-human primate species; however such viruses have never been fully characterized. The first simian homologue of HHV-6 was identified in common chimpanzees [217]. This virus was designated PanHV6 for *Pan troglodytes* herpesvirus 6. PanHV6 and HHV-6B have a 94–96% nucleotide and 95–97% amino acid sequence identity. Among 77 wild-caught chimpanzees, a unique PanHV6 strain was found in 21 animals. A roseolovirus was also identified in mandrill and drill monkeys, named MndHVβ (mandrill herpesvirus β) [218]. Most interestingly, HHV-6A infection was shown to accelerate the progression of AIDS (acquired immunodeficiency syndrome) in SIV (simian immunodeficiency virus)-infected macaques [211] and lead to neurological symptoms in marmosets [219].

The fact that African green monkeys, cynomolgus monkeys, pig-tailed monkeys, and marmosets can be infected experimentally with HHV-6 suggests that they are susceptible to human roseoloviruses (for review see [220]). In most cases, the animals remained clinically asymptomatic; only in rare cases, skin rash, fever, nasal discharge, splenomegaly, and lymphadenopathy were observed. Although HHV-6 did not replicate in mouse oligodendroglial precursor cells after in vitro infection, the transcription of some viral genes was observed in the mouse cells, and it caused cell cycle arrest [221]. Mice are naturally resistant to HHV-6 infection, possibly to their low sequence homology with human CD46, the cellular receptor of HHV-6. Mice transgenic for the human gene CD46 can be infected with HHV-6A, which established a long-term persistence in the brain along with leukocyte infiltrations [214].

## 7. Conclusions

Roseoloviruses are moving closer to the center of scientific and public interest. First of all, data has linked human roseoloviruses to several neurological diseases in the immunocompetent, including febrile seizures, MS, and Alzheimer’s disease. Secondly, HHV-6B infection in immunocompromised patients, particularly recipients of allogenic hematopoetic cell and solid organ transplants, causes clinically significant consequences. Currently, there is not a viable animal model for HHV-6B infection. However, the recent characterization of MRV revealed similar genetic and immunomodulatory characteristics to HHV-6B; MRV may be able to serve as a murine model for HHV-6B infection. Finally, PCMV/PRV may pose a risk in xenotransplantation that are close to a clinical application. Although it is still unclear whether PCMV/PRV can infect human cells, preclinical trials transplanting pig kidneys and hearts to baboons or cynomolgus monkeys clearly demonstrated that the presence of PCMV/PRV reduces the survival time of the xenotransplants significantly. Therefore detailed studies of roseoloviruses are important to understand their impact on the health of humans and animals.

Recently S. Chorlton performed a reanalysis of Alzheimer’s brain sequencing data reported by [113] and found an absence of purported HHV-6A and HHV-7 [222]. Additional studies are required to solve this controversy.

## Figures and Tables

**Table 1:** Genetic and biological properties of different roseoloviruses.

Property	HHV-6A	HHV-6B	HHV-7	MRV	PCMV/PRV
Natural host	Human	Human	Human	Mouse	Pig
Prevalence	Unknown	Greater than 90% of adults	Widespread	78% wild-caught house mice	Over 90% in a herd
Length of genome	159,322 bp	162,114 bp	153,080 bp	173,861 bp	128,367 bp
Genes encoding unique proteins	102	97	86	128	79
miRNA	4 predicted	4	Unknown	Unknown	Unknown
Initiation of replication	Origin-binding protein	Origin-binding protein	Origin-binding protein	Likely origin-binding protein	Unknown
Cell surface receptor	CD46	CD134	CD4	Unknown	Unknown
Cell tropism	CD4^+^ T cells UCBL^1^, PBMC^2^ CD8+ T cells NK cells	CD4+ T cells UCBL^1^, PBMC^2^	CD4^+^ T cells UCBL^1^, PBMC^2^	Likely thymocytes and/or thymic stromal cells	Lymphocytes
Transmission	Unknown	Saliva	Saliva	Saliva, breast milk	Body fluids
Susceptible T cell lines	SupT-1, HSB2, Jjhan	Molt-3, Mt-4, SupT-1	SupT-1	Unknown	Unknown
Replication in astrocytes	Productive	Low-level persistence	Unknown	Unknown	Unknown
Integration into cellular DNA and vertical transmission	Yes, into host telomeres	Yes, into host telomeres	Yes, needs confirmation	Unknown	Unknown
Disease associations in the natural host	Hashimoto’s thyroiditis Female infertility MS Encephalitis (rare) Alzheimer’s disease	Exanthem subitem febrile status epilepticus, febrile seizures, encephalitis	Exanthem subitem pityriasis rosea encephalitis (rare)	Asymptomatic T cell depletion, thymic atrophy, and failure to gain weight in infected neonates	Sub-clinical rhinitis, pneumonia, anemia, fever
Causes Immunosuppression	Yes	Yes	Unknown, but likely	Yes	Yes
Activated by immunosuppression	Yes	Yes	Unknown	Unknown	Possibly in xenotransplant recipient
Trans-species transmission	Marmoset (Callithrix jacchus): neurologic symptoms	Marmoset (Callithrix jacchus): Asymptomatic African green monkeys cynomolgus and pig-tailed macaques	Unknown	Unknown	Reduction of xenotransplant survival in non-human primates^3^

^1^ UCBL, umbilical cord blood lymphocytes; ^2^ PBMC, peripheral blood mononuclear cells, ^3^ unknown whether primates are infected.
